# Ecological and Health Risks Attributed to Rare Earth Elements in Coal Fly Ash

**DOI:** 10.3390/toxics12010071

**Published:** 2024-01-15

**Authors:** Latinka Slavković-Beškoski, Ljubiša Ignjatović, Mirjana Ćujić, Jelena Vesković, Katarina Trivunac, Jelena Stojaković, Aleksandra Perić-Grujić, Antonije Onjia

**Affiliations:** 1 Anahem Laboratory, Mocartova 10, 11160 Belgrade, Serbia; 2 Faculty of Physical Chemistry, University of Belgrade, Studentski trg 12-16, 11158 Belgrade, Serbia; 3 Vinča Institute of Nuclear Sciences, University of Belgrade, Mike Petrovića Alasa 12-14, 11351 Vinča, Serbia; 4 Faculty of Technology and Metallurgy, University of Belgrade, Karnegijeva 4, 11120 Belgrade, Serbia; 5 Innovation Center of the Faculty of Technology and Metallurgy, Karnegijeva 4, 11120 Belgrade, Serbia

**Keywords:** risk index, hazard quotient, cancer risk, multivariate analysis, Monte Carlo, REEs, REYs, power plant, heavy metal(loid)s, ICP-MS

## Abstract

The occurrence and distribution of yttrium and rare earth elements (REYs), along with major elements and heavy metal(loid)s (HMs) in coal fly ash (CFA) from five coal-fired power plants (CFPPs), were analyzed, and the REY-associated ecological and health risks were assessed. The individual REYs in CFA were abundant in the following order: Ce > La > Nd > Y > Pr > Gd > Sm > Dy > Er > Yb > Eu > Ho > Tb > Tm > Lu. The total REY content ranged from 135 to 362 mg/kg, averaging 302 mg/kg. The mean light-to-heavy REY ratio was 4.1, indicating prevalent light REY enrichment in CFA. Significantly positive correlations between the REYs suggested that they coexist and share similar origins in CFA. REYs were estimated to pose low to moderate ecological risks, with risk index (RI) values ranging from 66 to 245. The hazard index (HI) and target cancer risk (TCR) of REYs from CFA, estimated to be higher for children (HIc = 0.15, TCRc = 8.4 × 10^−16^) than for adults (HIa = 0.017, TCRa = 3.6 × 10^−16^), were well below the safety limits (HI = 1, TCR = 1.0 × 10^−6^). However, the danger to human health posed by HMs in the same CFA samples (HIc = 5.74, TCRc = 2.6 × 10^−4^, TCRa = 1.1 × 10^−4^) exceeded the safe thresholds (excl. HIa = 0.63). The mean RI and HI attributed to REYs in CFA were 14% and 2.6%, respectively, of the total risks that include HMs.

## 1. Introduction

Rare earth elements are the lanthanide group of chemical elements (Ce, Gd, La, Eu, Yb, Dy, Pr, Nd, Sm, Tb, Ho, Er, Tm, Lu, and Pm) plus Y (together referred to as REYs), which are typically found in the Earth’s crust at low concentrations [1,2]. REYs are significant because their unique magnetic, optical, catalytic, and electrical properties make them essential in producing advanced technologies, such as smartphones, electric vehicles, magnets, batteries, catalysts, radars, and missile guidance systems [1,3]. However, the ore deposits bearing REYs are widely dispersed and often poor in REY content, making these elements difficult to extract and refine economically [4].

They are primarily found in mineral deposits, such as bastnäsite, monazite, xenotime, and ion-adsorption clays [5,6]. These minerals are found in different geological formations, including igneous rocks, sedimentary rocks, and mineral sands. REYs are produced through a multi-step process that involves mining, leaching, extraction, and refining [7,8]. The specific methods vary depending on the deposit type and the concentration of REYs present.

Coal fly ash (CFA), a byproduct of coal burning in thermal power plants, has recently gained attention as a potential resource for REYs [9,10,11]. While challenges exist regarding low concentrations and extraction complexities since REYs are encapsulated within glassy particles [12], ongoing research and technological advancements are making it increasingly feasible to recover REYs from this unconventional source [13]. Further, this could contribute to more sustainable REY supply chains.

However, CFA poses a severe risk to both the environment [14,15,16] and human health due to its composition and potential for leaching [17,18,19,20,21] and dispersion [22,23,24]. The danger from CFA arises from the presence of various toxic substances, particularly heavy metal(loid)s (HMs), including As, Pb, Hg, Cd, Cr, and Ni [12,25]. These HMs are naturally present in coal and become concentrated in the ash during combustion. Exposure to HMs is dependent on their leachability, the size of the CFA particles, the environment, and meteorological conditions [26,27], and can lead to serious health issues, including neurological disorders, developmental problems in children, kidney damage, respiratory diseases, and even cancer [28].

Even though the content of REYs in CFA are relatively low compared to HMs, the toxicity of REYs induces doubt that the REY contribution to potential ecological and health risks may not be ignored [29]. Exposure to REYs has been linked to a range of health issues, including cytotoxicity to human cells [30], ischemic stroke [31], respiratory problems, kidney damage, and neurological disorders [32]. Therefore, it is crucial to assess the concentrations of REYs and HMs in CFA particles that could enter the environment and pose a risk to human health through various pathways.

There are many published articles about REY-associated risks. Ecological risk studies have been based on the toxicity coefficients of REYs, for which it was reported that they were similar to those of HMs [33]. It was reported that the ecological risks due to the REY presence in soil [34,35,36,37,38,39,40], sediment [41,42,43,44], and street dust were relatively low. Moreover, the estimated non-cancer and cancer human health risks from soil [45,46,47], sediments [48], air [49], and the consumption of vegetables fall below the acceptable safety limits [50,51].

However, there is a lack of a comprehensive evaluation of the risks from REYs in CFA. This study analyzes the occurrence, distribution, and ecological and health risks associated with REYs and HMs in CFA from different coal-fired power plants (CFPPs). Further, the Monte Carlo simulation (MCSs) enhanced the overall quality and reliability of the risk assessment process by running multiple simulations with randomly generated inputs of defined ranges to model a wide range of potential scenarios, accounting for the inherent uncertainties and variability [52]. Finally, this study also aims to measure the proportion of risks that the REYs contribute compared to those from HMs in the same CFA samples.

## 2. Materials and Methods

### 2.1. Samples, Chemicals, and Reagents

A total of 10 representative samples (5 bottom ash and 5 fly ash) were taken from five CFPPs, from different locations in Serbia (Figure S1). Fly ash samples (CFA1, CFA3, CFA5, CFA7, and CFA9) were collected from the CFPP-affiliated landfills, whereas the bottom ashes (CBA2, CBA4, CBA5, CBA8, and CBA10) were sampled directly from the boiler hoppers. A large stainless-steel sampling scoop was used to take ten individual samples that were merged into a composite sample. This sample was homogenized, and a portion of 0.5 kg was packed into an air-tight plastic (HDPE) container and transferred to the laboratory.

The REY and HM calibration solutions were prepared from mixed ICP-MS multi-element standards PE-MECAL2-ASL-1 (22 comps, 10 μg/mL each REY) and ICP-MS-6020-CAL-R-1 (22 comps, 10 μg/mL each HM) from Accustandard Inc. (New Haven, CT, USA). Single-element (Merck KGaA, Darmstadt, Germany) stock solutions for analytes Si, Al, Fe, and the internal standard ^115^In for ICP-MS were used. All other reagents and chemicals were purchased from Lachner s.r.o. (Neratovice, Czech Republic).

### 2.2. Chemical Analysis

Prior to the chemical analysis, the samples underwent drying (105 °C) and sieving (0.2 mm). A portion of the homogenized coal fly ash sample (0.1000 g) was weighed and placed in a platinum crucible. Then, 1.8 g lithium tetraborate and lithium metaborate fluxes were added to the sample in a 1:3 ratio. The mixture is then heated at 1050 °C for 60 min to fuse the sample. After cooling, the fused sample is dissolved in a dilute (5%) HNO_3_. The final solution is then analyzed using ICP-MS to determine the concentrations of the REYs, and ICP-OES to quantify Si, Al, and Fe.

In parallel, an accurately weighted 0.5 g of the homogenized CFA sample was digested with 7 mL of HNO_3_, 2 mL of HCl, and 1.0 mL of HF in a 100 mL Teflon tube at 185 °C in a microwave oven (model Mars 5, CEM Corporation, Matthews, NC, USA). When digestion is complete, the solution is transferred to a Teflon normal flask and diluted with 3% HNO_3_. This solution was used to measure the pseudo-total concentrations of HMs using ICP-MS.

An ICP-MS model iCAP Q and ICP-OES model iCAP 6500, both from Thermo Scientific Inc. (Waltham, MA, USA), were employed for the instrumental measurements. The operating conditions used in this study were described previously for ICP-OES [53] and ICP-MS [54].

The ICP-OES instrument operated in a radial view mode, with 1200 W forward RF power, argon gas, 12 L/min plasma coolant flow, 0.5 L/min auxiliary flow, and 50 rpm pump speed. The following wavelengths were used: Al (396.152), Fe (238.204), and Si (288.158).

A plasma power of 1550 W was set for the ICP-MS instrument. Argon gas flows were 13.8 L/min for cooling, 0.82 L/min for auxiliary, and 0.97 L/min for nebulization. Additionally, a helium gas flow of 5 mL/min was used in KED mode. The sample was introduced through a 2.5 mm internal diameter quartz injector, and nickel interface cones were employed. Data acquisition parameters included 20 sweeps per reading, 3 replicates, 3 points per peak, and dwell times ranging from 10 to 40 ms. The instrument operated in peak hopping scan mode with 30 sweeps. Sample flush time was set at 4 s, read delay time at 20 s, and wash time at 60 s. Matrix-matched external calibration was utilized for quantification. The isotopes (and their interferences) of each REE were as follows: ^45^Sc (COO, COOH), ^89^Y, ^139^La, ^140^Ce, ^141^Pr, ^146^Nd, ^147^Sm, ^153^Eu (BaO), ^157^Gd (CeOH, PrO), ^159^Tb (NdO), ^163^Dy (SmO), ^165^Ho (SmO), ^166^Er (SmO, NdO), ^169^Tm (SmO, EuO), ^172^Yb (GdO), ^175^Lu (GdO, TbO), and ^115^In.

Details on the method detection limits, calibration equations, linearity, recovery, and relative standard deviation (RSD) are given in Table S1.

### 2.3. Quality Assurance and Quality Control

The certified reference CFA material SRM 1633c from NIST (the National Institute of Standards and Technology, Washington, DC, USA) was used to check the accuracy of the analytical procedure. The recovery rates of 3 major elements (Fe, Al, and Si), 13 HMs (As, Cr, V, Pb, Ni, Mn, Ba, Cd, Cu, Mo, Co, Hg, and Zn), and 15 REYs (Ce, La, Y, Eu, Pr, Gd, Dy, Er, Yb, Ho, Tb, Nd, Sm, Tm, and Lu) were between 71% and 122%. Just to note, Pm was not included in this study since it is radioactive and has no stable isotopes. The samples and a reagent blank were run in five replicates to check for precision and background interference. The RSD of each element in replicate samples was ≤18%, and the linearity for all the elements was maintained at the calibration coefficients >0.996.

### 2.4. Normalization of REYs and Anomaly Calculation 

Normalization and anomaly calculation of the REYs in CFA can provide information regarding the fraction pattern, enrichment level, and provenance composition [7,41,55]. In this study, a Ce anomaly and Eu anomaly were used to reflect the geochemical parameters of REYs in the CFA. These anomalies were calculated using the following equations:

(1) $$Ce/Ce^{*}=Eu_{N}/\sqrt{Sm_{N}\times Gd_{N}}$$

(2) $$Eu/Eu^{*}=Ce_{N}/\sqrt{La_{N}\times Pr_{N}}$$

where *Ce*/Ce* and *Eu*/Eu* denote the Ce and Eu anomalies. The subscript *N* represents the NASC (North American Shale Composite)-normalized concentrations of REYs in CFA. The Ce and Eu anomalies can be positive (>1) or negative (<1).

### 2.5. Ecological Risk Indices

The impacts of lithogenic and anthropogenic activities were also evaluated using the ecological indices: enrichment factor (EF), geoaccumulation index (Igeo), and potential ecological risk index (RI) [56]. The equations and criteria for soil classification using ecological indices are given in Table S2 [57].

In this study, Mn is used as a reference element to estimate the EF values of REYs and HMs as follows:

(3) $$EF={\left(C_{x}/C_{Mn}\right)}_{sample}/{\left(C_{x}/C_{Mn}\right)}_{UCC}$$

where *C_x_* represents the concentration of individual element *x* in the CFA sample. The concentrations of REYs and HMs in the Upper Continental Crust (UCC) were used as the background values [58]. The CFA samples may be classified according to the EF values into five levels, from minimal (<2) to extremely high (≥40) enrichment. 

To evaluate the pollution by an element based on its background level, Igeo is calculated according to Equation (4):

(4) $$I_{geo}=log_{2}\left[C_{x}/\left(1.5\times C_{N}\right)\right]$$

where 1.5 is a constant, *C_x_* is the element concentration in the CFA sample, and *C_N_* is the element concentration in the reference (UCC) sample [58]. The classification is based on the Igeo index, which ranges from −5 to +5, and is given in Table S2.

The RI values classify a sample into four ecological risk levels from low (RI < 150) to very high (RI ≥ 600), and are based on the toxicity response (*T_r_*) coefficient of each HM (Hg = 40, Cr = 2, Cu = 5, Mn = 1, Ni = 5, Zn = 1, As = 10, Cd = 30, Co = 5, Pb = 5) [59] and REY (Ce = 1, La = 1, Y = 2, Nd = 2, Pr = 5, Sm = 5, Yb = 5, Dy = 5, Er = 5, Gd = 5, Ho = 10, Tb = 10, Tm = 10, Eu = 10, Lu = 20) [33]. Finally, RI is estimated as follows:

(5) $$RI=\sum_{i=1}^{n}T_{r}^{i}\times \left(C_{x}^{i}/C_{N}^{i}\right)$$

### 2.6. Human Exposure Assessment

Adverse human health effects of REYs and HMs in CFA for residents in the vicinity of CFPPs were assessed using the HRA model of the USEPA (the United States Environmental Protection Agency) [60,61]. Hazard risk (HI) and target cancer risk (TCR) indices were used to evaluate non-carcinogenic and carcinogenic risks to human health in two age groups (adults and children). All equations and the exposure factors in the model are given in Tables S3 and S4 [62]. Table S5 presents the reference dose (RfD) and cancer slope factor (CSF) for HMs, whereas the RfD of 0.02 mg kg^−1^ day^−1^ and the CSF of 3.2 × 10^−12^ were taken for REYs [36,47,63]. A worst-possible scenario was assumed for Cr, As, and Hg by taking the RfD and CSF values for Cr(VI), Hg(II), and As (inorganic).

### 2.7. Data Analysis

Basic calculations, descriptive statistics, and the Monte Carlo simulation of risks were performed using Microsoft Excel software (Microsoft Corp., San Francisco, CA, USA). Minitab software (Minitab LLC, State College, PA, USA) was used for statistical analyses, including normality (Ryan–Joiner) tests and correlation analysis. All plots were graphed using Microcal Origin (OriginLab Corp., Northampton, MA, USA).

## 3. Results and Discussion

### 3.1. Distribution of REYs in CFA

This study focused on characterizing the distribution of REYs in CFA from multiple locations. The Ryan–Joiner test indicated a normal data distribution. Table S6 presents the REY content in CFA samples (CFA1, CFA3, CF5, CFA7, and CFA9) from five CFPPs. 

The total REY content in these samples ranged from 135 to 362 mg/kg, averaging at 302 mg/kg. In parallel, coal bottom ash samples (CBA2, CBA4, CBA5, CBA8, and CBA10) from the same CFPPs were analyzed, showing notably lower REY contents (179 ± 55 mg/kg). Individual elements were found in the following descending order of abundance: Ce > La > Nd > Y > Pr > Gd > Sm > Dy > Er > Yb > Eu > Ho > Tb > Tm > Lu.

Furthermore, the light REYs (La, Ce, Pr, Nd, Pm, Sm, Eu, Gd) were the dominant, given the average light to heavy REYs (Dy, Ho, Tb, Tm, Yb, Lu, Er, Y) ratio of 4:1. From the economic aspect, the REYs were also divided into the critical (Eu, Tb, Nd, Dy, Y, Er) and excessive (Ce, Yb, Ho, Tm, Lu) REY groups [7]. Critical percentage and the outlook coefficient (C_outl_) are the two parameters that verify if CFA is worth recovering the REY or not [64]. Thus, if %Critical ≥ 30% and C_outl_ ≥ 0.7, the material can be considered a promising secondary source to recover the REY. All samples in this study meet these criteria.

CFA from Serbia was compared against other CFA from the European Union (EU), China, the United States (USA), South Africa (SA), the United Kingdom (UK), and India (Table 1). Disparities in REY content are attributed to variations in characteristics of the coal used, combustion conditions, and distinct procedures of ash treatment in the varying regions. Generally, CFA from Serbia has a slightly higher average REY content (302 mg/kg) than that reported from the EU (283 mg/kg), and has a significantly lower REY content than CFA from the USA (404 mg/kg) and China (473 mg/kg).

The Pearson correlation coefficients for REYs in CFA from CFPPs in Serbia indicate strong positive correlations between most REYs (Table S7). The correlation coefficients range from 0.806 (Tm vs. Pr) to 0.977 (La vs. Ce). These strong correlations suggest that the REYs have a mutual coexistence and likely share a common origin [70]. It highlights the potential for the simultaneous recovery of multiple REYs from CFA.

**Figure 1:**
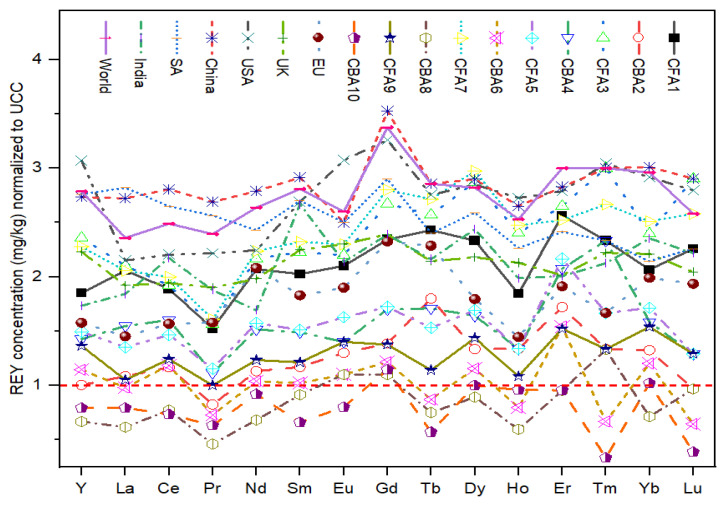
REY concentrations normalized to upper continental crust [58]. CFA samples 2, 4, 6, 8, and 10 are bottom ashes.

The UCC normalized REY concentrations are shown in Figure 1. These values range from 1 to 3 in most CFA samples, except for samples CFA10, CBA8, and partially CBA6. The maximum value (3.5) was obtained for Gd in an average sample from China, whereas the minimum (0.3) was recorded for Tm in the CFA10 sample.

**Figure 2:**
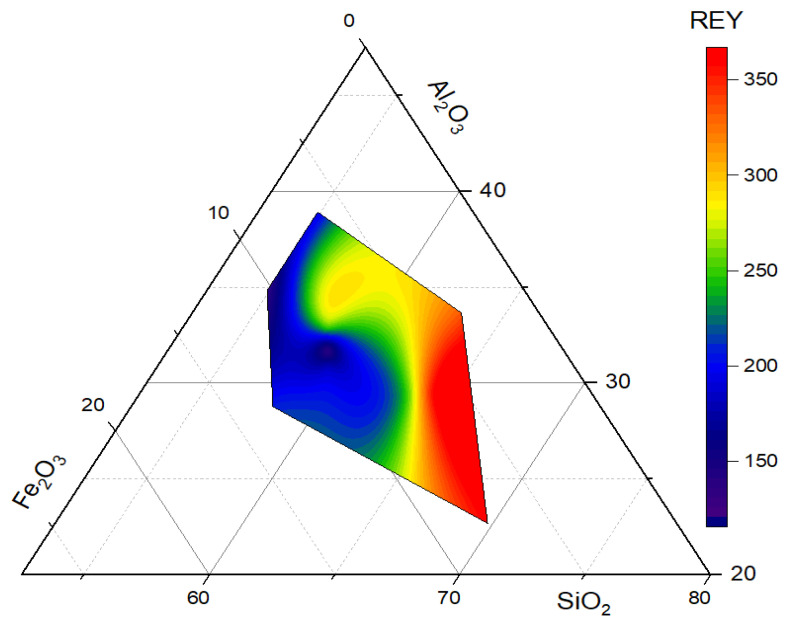
Ternery colormap surface showing the relationship between the REY content and major components in CFA.

Regardless of the source and composition of the coal being burned, CFA contains significant amounts of major components (SiO_2_, Al_2_O_3_, and Fe_2_O_3_) [71]. Figure 2 shows that the content of these components of CFA has a positive effect on the REY content. The presence of SiO_2_, Al_2_O_3_, and Fe_2_O_3_ in CFA provides a suitable environment for REYs and promotes their accumulation. Thus, SiO_2_ is a binder, holding the different REYs together in the ash matrix, while Al_2_O_3_ acts as a stabilizing agent, helping to retain the REYs and prevent their leaching into the environment. Additionally, fluctuations in redox conditions within CFA can alter the oxidation states of Fe and REYs, thereby impacting their behavior in the ash and enabling Fe_2_O_3_ to serve as a carrier for certain REYs. Therefore, this implies that the levels of major mineral components can act as an indicator of the presence and quantity of REYs in CFA.

The Ce and Eu anomalies in the REY content in CFA are presented in Table 2. The Ce anomaly values range from 0.93 to 1.16, with an average of 1.03. This means that the Ce content is generally close to the average content of the other REYs in CFA. The Eu anomaly values, on the other hand, vary from 1.09 to 1.57, with an average value of 1.29. This indicates that the Eu concentration in CFA tends to deviate more from the average concentration of the other REYs. CBA6 and CBA8 demonstrate notably high Eu anomalies, suggesting an enrichment of Eu in these ashes.

Conversely, CFA10 displays a lower Eu anomaly of 1.12, indicating a potential depletion of Eu. It may be assumed that the Eu variation is more pronounced in bottom ash since these samples belong to this ash type. An anomaly variation is seen when comparing the Ce and Eu anomalies between different countries. The Ce anomaly values range from 0.82 (China) to 1.16 (CBA8). This means that the Ce concentration in CFA is slightly higher than the average concentrations of the other REYs worldwide. In contrast, the Eu anomaly values span from 1.07 (SA) to 1.57 (CBA8), with the world average being 1.13. The Eu concentration in CFA varies significantly across different countries, with Serbia having the highest deviation from the average.

### 3.2. Potential Ecological Risk of REYs in CFA

The ecological risk of REYs in CFA was analyzed by evaluating the EF, I_geo_, and RI indices. These indices provide insights into the degree of REY accumulation in CFA compared to UCC background and the potential ecological risks associated with it.

The EF values (Table S8) for individual REYs in different CFA samples (CFA1 to CFA10) vary across the elements. Thus, CFA7 shows relatively high EF values for most REY, whereas CFA10 exhibits very low EF values.

**Figure 3:**
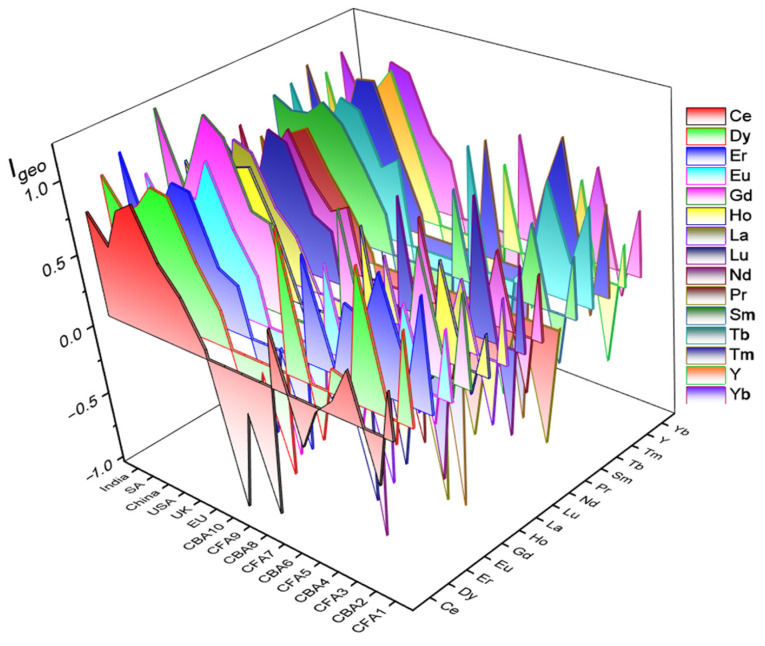
Geoaccumulation index (I_geo_) for REYs in CFA.

Next, the results of the I_geo_ are displayed in Figure 3 and Table S9. A diversity of I_geo_ values is recorded, with positive I_geo_ values for most REYs in fly ash samples, indicating moderate to significant REY accumulation. In contrast, bottom ash samples (CBA2, CBA4, CBA6, CBA8, CBA10) show negative values. Moreover, CFA10 consistently exhibits strongly negative I_geo_ values across multiple REYs. A higher I_geo_ in fly ash than bottom ash confirms that REYs are more enriched in this ash fraction. This implies that besides the mineralogy of the coal, the REY content is influenced by the combustion process.

Furthermore, the distribution of REYs in CFA follows a trend of light REY < heavy REY, which means that the enrichment of REY increases with atomic number. Comparing the I_geo_ values of CFA with those of different regions, it can be observed that CFA from the USA and China generally exhibit higher accumulation levels than CFA from the EU, UK, India, and South Africa. The I_geo_ values for REYs in CFA from the world as a whole range from −2.17 for Tm in sample CF10 to 1.23 for Gd in an average sample from China.

**Figure 4:**
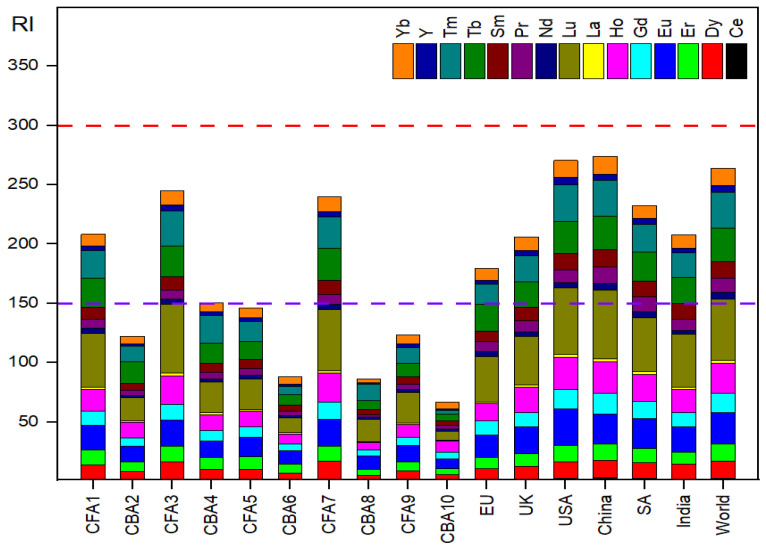
Potential ecological risk index (RI) values for individual REYs in CFA.

The outcome of the RI calculations suggested a low to moderate ecological risk presented by REYs in Serbia, with values ranging from 66 to 245, with an average RI value of 148. However, the RI of REYs falls into moderate ecological risk (150 ≤ RI ≤ 300) for all fly ash samples worldwide (Figure 4). The individual risk index (E_r_) values of various REYs in CFA are quite different. Specifically, Lu shows the highest RI value of 58.1 in CFA3 and the China average, followed by Lu with an RI of 55.9 in the USA average. The lowest E_r_ values are observed in CBA8 for La and CFA10 for Ce, with values of 0.6 and 0.7, respectively.

In the context of global comparison, the RI values for the REYs in CFA from various countries (EU, UK, USA, China, SA, India) and the world average reveal that the ecological risk varies geographically, and China and the USA exhibit higher RI values compared to other regions.

In parallel to the evaluation of RI from REYs, HMs were analyzed, and the corresponding RI was calculated (Table S10). Unlike REYs, HMs are found in CFA at a significantly higher level; therefore, the risk arising from them is significantly higher. CFA5 poses the highest RI of 1740, primarily due to highly elevated concentrations of Cd (2.7 mg/kg) and As (329 mg/kg). This indicates a potentially high ecological risk associated with these HMs in CFA7. In contrast, CFA10 has the lowest RI of 598, which is attributed to comparatively lower concentrations of the assessed HMs. Among the HMs, As, Cd, and Hg contribute mostly to the overall RI values in all samples. Generally, the results show that the composition of HMs in CFA varies, and certain samples pose a higher ecological risk than others. The mean RI value, which includes REYs and HMs in CFA, amounts to 907.

**Figure 5:**
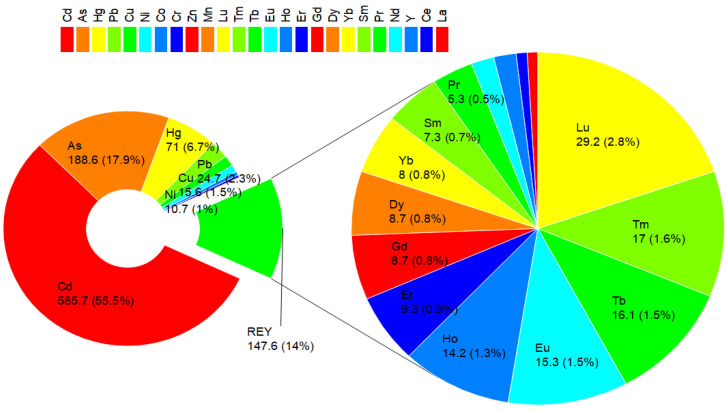
Contribution of REYs and HMs to risk index (RI) from CFA.

In conclusion, the ecological risks caused by REYs in CFA were relatively low compared to those of HMs in CFA. The mean RI value attributed to REYs accounted for 14% of the total risk posed by REYs and HMs. In terms of individual REYs (Figure 5, Table S11), Lu has the highest E_r_ value (29.2), while Pr has the smallest one (5.3).

### 3.3. Human Health Risk Assessment 

Harmful health effects of REY in CFA were evaluated using the USEPA model for residential receptors, including children and adults. This model has taken into account all potential exposure pathways (inhalation, ingestion, and dermal contact). The obtained HQ values for both age groups decreased in the order as follows (min–max): Ce (0.00318–0.08078) > La (0.00131–0.04176) > Nd (0.00127–0.03871) > Y (0.00096–0.03178) > Dy (0.00024–0.00744) > Pr (0.00022–0.00732) > Gd (0.00030–0.00718) > Sm (0.00021–0.00699) > Er (0.00015–0.00391) > Yb (0.00010–0.00315) > Eu (0.00006–0.00147) > Ho (0.00003–0.00132) > Tb (0.00003–0.00122) > Lu (0.00001–0.00058) > Tm (0.00001–0.00058). The mean HQ values for REYs in CFA are presented in Table S12. Based on the HQ values of REYs, it may be concluded that REYs have a negligible adverse effect on humans.

**Figure 6:**
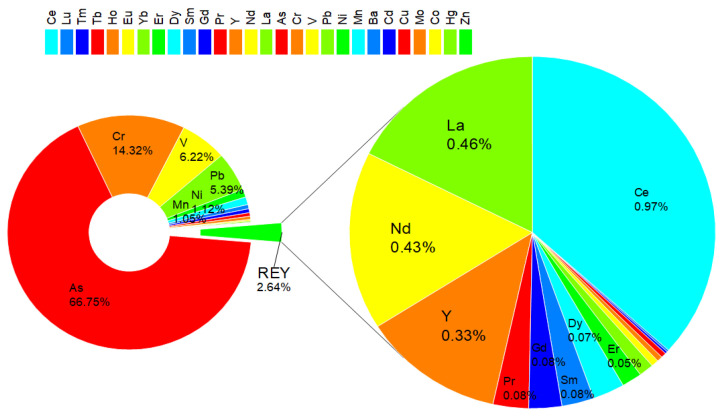
Contribution of REYs and HMs to hazard index (HI) from CFA.

However, the human health risks attributed to HMs in the same CFA samples exceeded the safe limits for non-carcinogenic risk (HI = 1) for children and cancer risk (TCR = 1 × 10^−4^) for both age groups (Table 3). Additionally, HI > 1 results were obtained for HIa and HIc for sample CFA5. At the same time, the mean TCR values for adults and children are 1.1 × 10^−4^ and 2.6 × 10^−4^, respectively. It should be pointed out that the HI and TCR values associated with HMs are much higher than those of REYs (Figure 6).

The HQ and TCR values for As are the highest, with the mean HQ of 4.2 × 10^−1^ for HIa and 3.9 for HIc (Table S13), and 3.8 × 10^−5^ for CRa and 1.5 × 10^−4^ for CRc (Table S14). This is followed by Cr with mean HQ values of 9.0 × 10^−2^ for HIa and 7.9 × 10^−1^ for HIc, and 2.6 × 10^−5^ for CRa and 9.9 × 10^−^^5^ for CRc. The other HMs have lower HQ and CR values. It is noteworthy that even low HQ values can be concerning, particularly in the case of long-term exposure or when considering the cumulative effects of multiple HMs.

In any case, it can be concluded that the adverse effect of fly ash on human health originates to a much greater extent from the presence of HMs than REYs. Namely, the mean value of HI from REYs is only 2.6% of the total HI, in which the share of HI related to HMs is as much as 97.2%.

**Figure 7:**
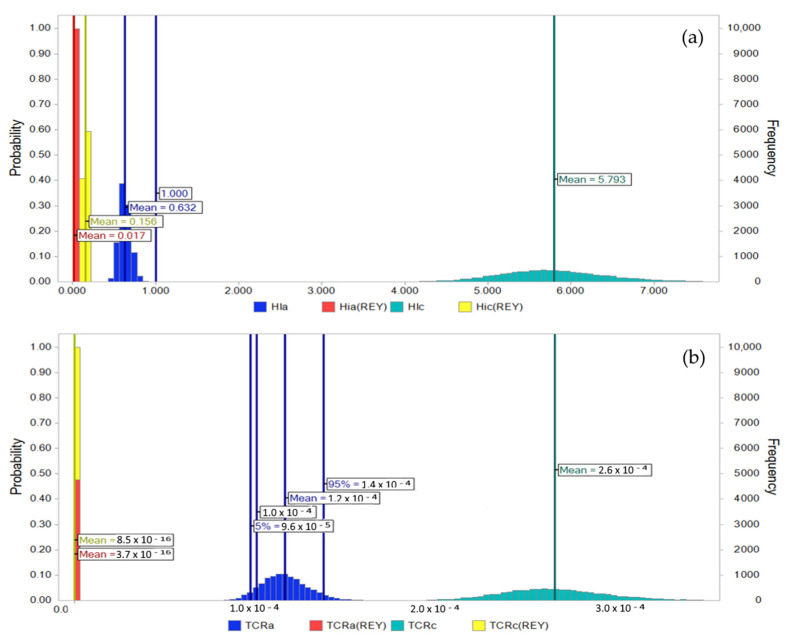
Monte Carlo simulation of the hazard index (HI) (**a**) and target cancer risk (TCR) (**b**) of REYs and HMs in CFA.

Because single-point deterministic estimates of risks fail to account for uncertainty and variability, MCS was applied to HI and TCR evaluation (Figure 7). The MCS method generates probabilistic distributions of HI and TCR, providing insights into a range of health risks that children and adults could be subjected to with varying degree of certainty. Based on the MCS results, the mean values for HI and TCR attributed to REYs and the total HIa remain vastly below safety thresholds. Unfortunately, the total HIc and TCRc, including both REYs and HMs, far exceed the save limits. In contrast, the mean value for TCRa is 1.2 × 10^−4^, while the 5% percentile is in the area below 1 × 10^−4^. In all cases, the TCR values were well above the 1 × 10^−6^ limit. According to the sensitivity analysis of MCS, body weight (BW), ingestion rate (IR), and exposure frequency (EF) are the three most sensitive exposure factors for the health risk variability of CFA in children and adults.

## 4. Conclusions

REYs in CFA from CFPPs in Serbia were analyzed, including concentration distribution, correlation, ecological indices, and non-carcinogenic and carcinogenic human health risks. The highest average concentration of total REYs was present in CFA7 (362 mg/kg), followed by CFA3 (356 mg/kg) and CFA1 (333 mg/kg). The values of LREY/HREY ratios ranged from 3.6 to 4.3. All REYs were strongly correlated. Two REYs (Ce and La) have significantly higher concentrations than the other REYs. The average Ce and Eu anomalies were 1.03 and 1.29, respectively. REYs in fly ash pose a moderate ecological risk, but the total risk of CFA, which includes HMs, is severe. In this case, only 14% of RI, on average, was attributed to REYs. A vast majority of RI from CFA was attributed to Cd, As, and Hg. The most RI-influencing REY is Lu, with 2.8% of the RI share. Even though REYs in CFA exhibit negligible risks to human health, CFA is carcinogenic for humans and poses an unacceptable non-carcinogenic risk for children, dominantly due to the presence of HMs. REYs contribute less than 3% to total HI, with Ce as the main contributor. According to the MCS sensitivity analysis, IR, BW, and EF mostly influence the risk variability.

## Figures and Tables

**Table 1:** REY concentrations ^#^ (mg/kg) in CFA samples (mean ± SD) compared with those reported worldwide.

REY	Serbia ^#^	Serbia ^#,^*	EU	UK	USA	China	SA	India	World
Ce	107	69	99	122	139	177	167	137	157
Dy	8.8	4.7	7.0	8.5	11	11	10	10	11
Er	5.3	3.3	4.4	4.6	6.4	6.5	5.6	4.6	6.9
Eu	1.9	1.1	1.9	2.3	3.1	2.5	2.5	2.1	2.6
Gd	8.7	5.2	9.3	9.6	13	14	12	10	14
Ho	1.5	0.8	1.2	1.8	2.3	2.2	1.9	1.7	2.1
La	53	31	45	60	67	84	87	57	73
Lu	0.6	0.3	0.6	0.6	0.9	0.9	0.7	0.7	0.8
Nd	50	29	56	53	61	75	66	46	71
Pr	9.7	5.3	11	14	16	19	18	13	17
Sm	8.7	4.9	8.6	11	13	14	13	13	13
Tb	1.5	0.8	1.6	1.5	1.9	2.0	1.7	1.5	2.0
Tm	0.7	0.4	0.5	0.7	0.9	0.9	0.7	0.6	0.9
Y	39	21	33	47	64	57	58	36	59
Yb	4.0	2.3	3.9	4.3	5.7	5.9	4.2	4.6	5.8
∑LREY	240	145	231	271	311	386	365	277	347
∑HREY	62	34	52	69	94	87	83	60	88
∑Critical	107	60	104	117	148	155	143	100	152
%Critical	35	33	37	34	37	33	32	30	35
Outlook	0.94	0.82	0.99	0.90	0.99	0.83	0.82	0.69	0.91
∑REY	302	179	283	340	404	473	448	337	435
Reference	[**]	[**]	[65]	[66]	[67]	[7]	[68]	[13]	[69]

^#^—mean values calculated from data in Table S6; *—bottom ash; **—this study.

**Table 2:** Ce and Eu anomalies in CFA.

CFA, CBA	Ce Anomaly	Eu Anomaly
CFA1	1.02	1.15
CBA2	1.08	1.33
CFA3	0.95	1.15
CBA4	0.93	1.32
CFA5	1.07	1.27
CBA6	1.04	1.52
CFA7	0.95	1.19
CBA8	1.16	1.57
CFA9	1.14	1.31
CBA10	0.97	1.12
EU	0.97	1.12
UK	1.05	1.10
USA	1.10	1.09
China	0.82	1.12
SA	0.94	1.07
India	0.89	1.26
World	0.89	1.13

**Table 3:** Hazard index (HI) and target cancer risk (TCR) for REYs and REYs + HMs in CFA.

CFA, CBA	HIa (REY + HMs)	HIa (REY)	HIc (REY + HMs)	HIc (REY)	TCRa (REY + HMs)	TCRa (REY)	TCRc (REY + HMs)	TCRc (REY)
CFA1	0.56	0.02	5.12	0.21	1.0 × 10^−4^	5.0 × 10^−16^	2.3 × 10^−4^	1.2 × 10^−15^
CBA2	0.51	0.01	4.68	0.12	1.1 × 10^−4^	2.9 × 10^−16^	2.5 × 10^−4^	6.7 × 10^−16^
CFA3	0.62	0.02	5.69	0.23	1.2 × 10^−4^	5.4 × 10^−16^	2.7 × 10^−4^	1.3 × 10^−15^
CBA4	0.49	0.02	4.42	0.17	1.1 × 10^−4^	3.9 × 10^−16^	2.5 × 10^−4^	9.2 × 10^−16^
CFA5	1.71	0.02	15.9	0.16	2.7 × 10^−4^	3.8 × 10^−16^	6.2 × 10^−4^	8.8 × 10^−16^
CBA6	0.46	0.01	4.21	0.12	8.3 × 10^−5^	2.8 × 10^−16^	1.9 × 10^−4^	6.5 × 10^−16^
CFA7	0.86	0.02	7.96	0.23	1.5 × 10^−4^	5.5 × 10^−16^	3.3 × 10^−4^	1.3 × 10^−15^
CBA8	0.39	0.01	3.54	0.08	8.7 × 10^−5^	1.8 × 10^−16^	2.0 × 10^−4^	4.3 × 10^−16^
CFA9	0.36	0.01	3.27	0.13	6.3 × 10^−5^	3.1 × 10^−16^	1.4 × 10^−4^	7.3 × 10^−16^
CBA10	0.29	0.01	2.60	0.09	5.2 × 10^−5^	2.0 × 10^−16^	1.2 × 10^−4^	4.7 × 10^−16^
Mean HI	0.63	0.017	5.74	0.15	1.1 × 10^−4^	3.6 × 10^−16^	2.6 × 10^−4^	8.4 × 10^−16^

## Data Availability

The data presented in this study are available on request from the corresponding author.

## Supplementary Materials

The following supporting information can be downloaded at: https://www.mdpi.com/article/10.3390/toxics12010071/s1, Figure S1: Map of sampling locations; Table S1: Validation parameters of REY, HMs, and major elements in CFA samples using ICP-MS. The method detection limit (MDL), calibration equation, correlation coefficient (r^2^), recovery (R), and relative standard deviation; Table S2: Ecological risk indices, the equations, and classification [53]; Table S3: Equations used in the risk assessment model [58]; Table S4: Exposure parameters and their distributions in the risk assessment model [58]; Table S5: Reference doses (RfD) (mg·kg^−1^·day^−1^) and the cancer slope factors (CSF) (kg·day·mg^−1^) of HMs for ingestion, inhalation, and dermal pathway [58]; Table S6: REY concentrations (mg/kg) in CFA samples from coal-fired power plants in Serbia (mean ± SD); Table S7: Pearson correlation coefficients for the REY content in CFA samples from coal-fired power plants in Serbia; Table S8: Enrichment factor (EF) for REY in CFA; Table S9: Geoaccumulation index (Igeo) for REY in CFA; Table S10: Potential risk index (RI) for HMs in CFA; Table S11: Individual risk index (Er) for HMs and REY in CFA; Table S12: Mean hazard quotients (HQ) for REY in CFA; Table S13: Mean hazard quotients (HQ) for HMs in CFA; Table S14: Individual mean cancer risk (CR) for HMs in CFA.
