# Supplementary material for: Ecological and Health Risks Attributed to Rare Earth Elements in Coal Fly Ash
Source: Toxics. 2024 Jan 15;12(1):71. doi: 10.3390/toxics12010071 (PMC10818428; doi:10.3390/toxics12010071)
Supplement: Supplementary file 1 [file toxics-12-00071-s001.zip › toxics-2788513-supplementary.pdf]

Supplementary Materials: Ecological and Health Risks Attributed to Rare Earth Elements in Coal Fly Ash

Latinka Slavković-Beškoski, Ljubiša Ignjatović, Mirjana Ćujić, Jelena Vesković, Katarina Trivunac, Jelena Stojaković, Aleksandra Perić-Grujić, Antonije Onjia

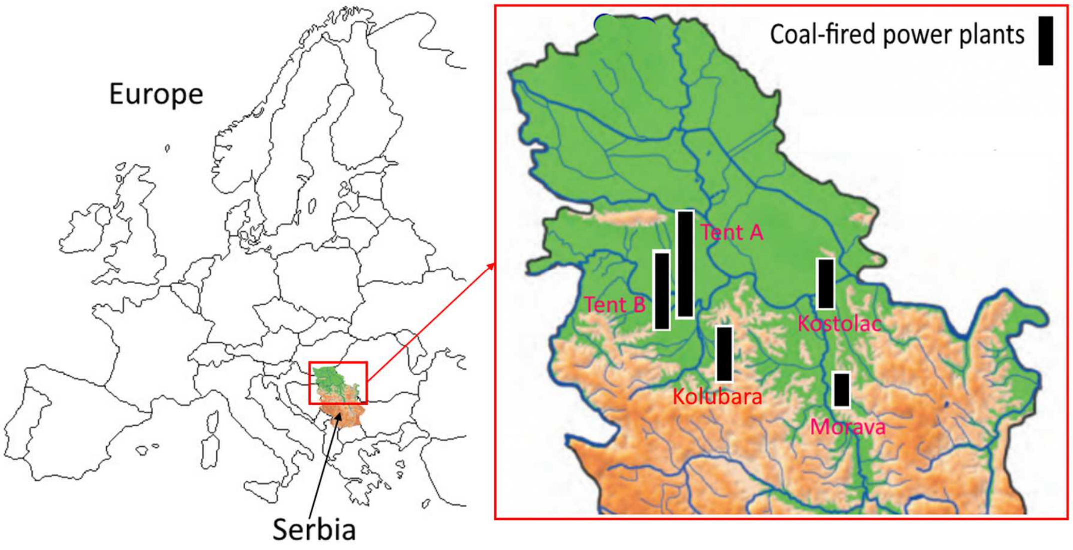

Figure S1. Map of sampling locations.

Table S1. Validation parameters of REY, HMs, and major elements in CFA samples using ICP-MS. The method detection limit (MDL), calibration equation, correlation coefficient (r^2), recovery (R), and relative standard deviation (RSD).

Element	A+B·x	r^2	MDL (mg/kg)	R (%)	RSD (%)	NIST 1633c (mg/kg)
^{140}Ce	229 + 331929·x	0.9994	0.005	87	9.2	180
^{163}Dy	3.41 + 143402·x	0.9997	0.007	81	9.0	18.70
^{166}Er	17.2 + 219498·x	0.9996	0.006	76	15	***
^{153}Eu	3.42 + 230142·x	0.9996	0.008	92	7.8	4.67
^{157}Gd	3.44 + 120166·x	0.9996	0.007	93	9.8	***
^{165}Ho	7.23 + 650547·x	0.9999	0.006	71	18	***
^{139}La	51.3 + 259834·x	0.9996	0.005	122	8.9	87.0
^{175}Lu	1.09 + 428953·x	0.9998	0.016	71	18	1.32
^{146}Nd	9.87 + 69876·x	0.9995	0.009	113	10	87
^{141}Pr	7.08 + 349674·x	0.9997	0.005	92	8.7	***
^{147}Sm	4.11 + 70127·x	0.9996	0.010	89	14	19
^{159}Tb	3.22 + 597865·x	0.9999	0.006	91	12	3.12
^{169}Tm	1.30 + 701623·x	0.9998	0.012	71	15	***
^{172}Yb	1.17 + 201213·x	0.9996	0.011	74	16	7.7
^{89}Y	687 + 81024·x	0.9991	0.014	85	11	***
^{75}As	11.2 + 3442·x	0.9976	0.019	86	13	186.2
^{52}Cr	318 + 2979·x	0.9992	0.063	79	5.2	258
^{51}V	164 + 11782x	0.9994	0.021	86	14	286.2
^{206}Pb	227 + 12912·x	0.9993	0.041	97	7.5	95.2
^{60}Ni	183 + 2710·x	0.9991	0.044	114	10	132
^{55}Mn	43.6 + 3541·x	0.9989	0.059	94	9.7	240.2
^{137}Ba	664 + 1492·x	0.9978	0.077	82	14	1126
^{111}Cd	33.3 + 6169·x	0.9996	0.018	92	10	0.758
^{63}Cu	295 + 6788·x	0.9999	0.092	82	9.2	173.7
^{98}Mo	157 + 12501·x	0.9997	0.032	108	4.2	***
^{59}Co	226 + 2847·x	0.9996	0.047	91	7.7	42.9
^{202}Hg	76.9 + 1102·x	0.9966	0.081	118	17	1.005
^{66}Zn	89.7 + 3876·x	0.9987	0.103	92	11	235
* Si	393 + 5362·x	0.9967	0.142	86	16	21.30**
* Al	88.1 + 4489·x	0.9995	0.138	104	9.6	13.28**
* Fe	118 + 9012·x	0.9981	0.171	93	8.4	10.49**

* - measured by ICP-OES; ** - concentration in %; *** – not available in SRM NIST 1633c (spiked sample was used for the recovery and RSD tests.

Table S2. Ecological risk indices, the equations, and classification [53].

Index,<br>Value	Equation,<br>Class
Enrichment factor	$EF = [(C_x / R)_{sample}] / [(C_x / R)_{reference}]$
EF < 2	- deficiency to minimal enrichment
2 ≤ EF < 5	- moderate enrichment
5 ≤ EF < 20	- significant enrichment
20 ≤ EF < 40	- very high enrichment
EF ≥ 40	- extremely high enrichment.
Geoaccumulation index	$I_{geo} = \log_2 [C_n / (1.5 \times B_n)]$
$I_{geo} < 0$	- Unpolluted
$0 < I_{geo} < 1$	- unpolluted to moderately polluted
$1 \leq I_{geo} < 2$	- moderately polluted
$2 \leq I_{geo} < 3$	- moderately to strongly polluted
$3 \leq I_{geo} < 4$	- strongly polluted
$4 \leq I_{geo} < 5$	- strongly to extremely polluted
$I_{geo} \geq 5$	- extremely polluted.
Risk index	$RI = \sum (T_i^i \times C_{is}^i / C_{in}^i)$
RI < 150	- low ecological risk
150 ≤ RI < 300	- moderate ecological risk
300 ≤ RI < 600	- significant ecological risk
RI ≥ 600	- very high ecological risk.

Table S3. Equations used in the risk assessment model [58].

Average Daily Dose

$$ADD_{ing} = \frac{C \times IngR \times EF \times ED}{BW \times AT} \times CF$$

$$ADD_{inh} = \frac{C \times InhR \times EF \times ED}{PEF \times BW \times AT}$$

$$ADD_{derm} = \frac{C \times SA \times AF \times ABS \times EF \times ED}{BW \times AT} \times CF$$

Non-Carcinogenic Risk

$$HQ_{i,inh} = \frac{ADD_{i,inh}}{RfD_{i,inh}}$$

$$HQ_{i,derm} = \frac{ADD_{i,derm}}{RfD_{i,derm}}$$

$$HQ_i = HQ_{i,ing} + HQ_{i,inh} + HQ_{i,derm}$$

$$HI = \sum_{i=1}^{n} HQ_i$$

Carcinogenic Risk

$$CR_{i,ing} = CDI_{i,ing} \times CSF_{i,ing}$$

$$CR_{i,inh} = CDI_{i,inh} \times CSF_{i,inh}$$

$$CR_{i,derm} = CDI_{i,derm} \times CSF_{i,derm}$$

$$CR_i = CR_{i,ing} + CR_{i,inh} + CR_{i,derm}$$

$$TCR = \sum_{i=1}^{n} CR_i$$

The risk assessment model parameters: C (element concentration), IngR (ingestion rate), InhR (inhalation rate), EF (exposure frequency), ED (exposure duration), PEF (particulate emission factor), SA (skin surface area), AF (adherence factor), ABS (dermal absorption factor), BW (body weight), ATnc (average non-carcinogenic time), ATc (average carcinogenic time), CF (conversion factor), LT (lifetime), RfD (reference dose), CSF (cancer slope factor).

Table S4. Exposure parameters and their distributions in the risk assessment model [58].

Exposure parameter	Abbrev.	Units	Children	Adults	Distribution
Element concentration	C	$mg·kg^{-1}$	-	-	Lognormal
Ingestion rate	IngR	$mg·day^{-1}$	200	100	Triangular
Inhalation rate	InhR	$m^3·day^{-1}$	7.6	20	Lognormal
Exposure frequency	EF	$day(s)·year^{-1}$	350	350	Triangular
Exposure duration	ED	year(s)	6	24	Point
Particulate emission factor	PEF	$m^3·kg^{-1}$	1.36×10^9	1.36×10^9	Point
Skin surface area	SA	cm^2	2800	5700	Point
Adherence factor	AF	$mg·cm^{-2}·day^{-1}$	0.2	0.07	Lognormal
Dermal absorption factor	ABS	unitless	0.001	0.001	Point
Body weight	BW	kg	15	70	Lognormal
Average non-carcinogenic time	AT_{nc}	day(s)	2190	8760	Point
Average carcinogenic time	AT_c	day(s)	25,550	25,550	Point
Conversion factor	CF	$kg·mg^{-1}$	10^{-6}	10^{-6}	Point
Lifetime	LT	year(s)	70	70	Point
Reference dose	RfD	$mg·kg^{-1}·day^{-1}$	**	**	Point
Cancer slope factor	CSF	$kg·day·mg^{-1}$	**	**	Point

Table S5. Reference doses (RfD) ($mg \cdot kg^{-1} \cdot day^{-1}$) and the cancer slope factors (CSF) ($kg \cdot day \cdot mg^{-1}$) of HMs for ingestion, inhalation, and dermal pathway [58].

Element	RfD_{ing}	RfD_{inh}	RfD_{derm}	CSF_{ing}	CSF_{inh}	CSF_{derm}
As (inorganic)	3×10^{-4}	3×10^{-4}	1.23×10^{-4}	1.5	15.1	3.66
Ba	2×10^{-1}	-	-	-	-	-
Cd	1×10^{-3}	1×10^{-5}	1×10^{-5}	6.1	6.3	6.1
Co	2×10^{-2}	-	1.6×10^{-2}	-	-	-
Cr(III)	1.5	-	-			
Cr(VI)	3×10^{-3}	2.86×10^{-5}	6×10^{-5}	0.5	42	20
Cu	4×10^{-2}	4.02×10^{-2}	1.2×10^{-2}	-	-	-
Fe	7×10^{-1}	-	-	-	-	-
Hg(II)	3×10^{-4}	8.6×10^{-5}	2.1×10^{-5}	-	-	-
MeHg	1×10^{-4}	-	-	-	-	-
Mn	1.4×10^{-1}	-	1.84×10^{-3}	-	-	-
Mo	5×10^{-3}	-	-	-	-	-
Ni	2×10^{-2}	2.06×10^{-2}	5.4×10^{-3}	-	-	-
Pb	3.5×10^{-3}	3.52×10^{-3}	5.25×10^{-4}	0.0085	0.042	-
Sb	4×10^{-4}	-	-	-	-	-
V	7×10^{-3}	7×10^{-3}	7×10^{-5}	-	-	-
Zn	3×10^{-1}	3×10^{-1}	6×10^{-2}	-	-	-

Table S6. REY concentrations (mg/kg) in CFA samples from coal-fired power plants in Serbia (mean±SD).

REY	CFA1 (Tent A)	CBA2 (Tent A*)	CFA3 (Tent B)	CBA4 (Tent B*)	CFA5 (Morava)	CBA6 (Morava*)	CFA7 (Kolubara)	CBA8 (Kolubara*)	CFA9 (Kostolac)	CBA10 (Kostolac*)
Ce	119±11	74±6.8	121±11	101±9.2	92±8.5	75±6.9	126±12	49±4.5	78±7.2	46±4.3
Dy	9.1±0.8	5.2±0.5	11.2±1	6.4±0.6	6.6±0.6	4.5±0.4	11.6±1.0	3.5±0.3	5.6±0.5	3.9±0.4
Er	5.9±0.9	4.0±0.6	6.1±0.9	4.8±0.7	5±0.7	3.5±0.5	5.8±0.9	2.2±0.3	3.5±0.5	2.2±0.3
Eu	2.1±0.2	1.3±0.1	2.2±0.2	1.4±0.1	1.6±0.1	1.1±0.1	2.3±0.2	1.1±0.1	1.4±0.1	0.8±0.1
Gd	9.4±0.9	5.5±0.5	10.7±1	6.8±0.7	6.9±0.7	4.9±0.5	11.2±1.1	4.4±0.4	5.5±0.5	4.6±0.4
Ho	1.5±0.3	1.1±0.2	2.1±0.4	1.1±0.2	1.0±0.2	0.66±0.12	2.1±0.4	0.5±0.09	0.9±0.16	0.8±0.14
La	64±5.7	34±3.0	65±5.8	48±4.3	42±3.7	30±2.7	64±5.7	19±1.7	33±2.9	25±2.2
Lu	0.7±0.13	0.3±0.05	0.9±0.16	0.4±0.07	0.4±0.07	0.2±0.04	0.8±0.14	0.3±0.05	0.4±0.07	0.1±0.02
Nd	56±5.4	31±2.9	59±5.6	41±3.9	43±4.1	28±2.7	60±5.8	18±1.8	33±3.2	25±2.4
Pr	10.8±0.9	5.9±0.5	11.1±1.0	7.9±0.7	8.2±0.7	5.2±0.4	11.4±1.1	3.3±0.3	7.1±0.6	4.5±0.4
Sm	9.5±1.3	5.5±0.8	10.5±1.5	7.0±1.2	7.1±1.1	4.8±0.7	10.9±1.5	4.3±0.6	5.7±0.8	3.1±0.4
Tb	1.7±0.2	1.2±0.15	1.8±0.22	1.2±0.14	1.07±0.13	0.61±0.07	1.9±0.23	0.53±0.06	0.8±0.1	0.4±0.05
Tm	0.7±0.11	0.4±0.06	0.9±0.14	0.7±0.11	0.5±0.08	0.2±0.03	0.8±0.12	0.4±0.06	0.4±0.06	0.1±0.02
Y	39±4.3	21±2.3	50±5.5	30±3.3	31±3.5	24±2.6	48±5.3	14±1.5	29±3.2	17±1.8
Yb	4.1±0.6	2.6±0.4	4.7±0.8	3.1±0.5	3.4±0.5	2.4±0.4	4.9±0.8	1.4±0.2	3±0.5	2±0.3
ΣLREY	270	156	279	213	201	149	286	99	164	109
ΣHREY	63	36	77	48	49	36	76	23	43	26
ΣCritical	114	63	129	85	88	62	130	40	73	49
%Critical	34	33	36	32	35	33	36	33	35	36
Outlook	0.90	0.81	1.00	0.80	0.90	0.79	0.96	0.78	0.88	0.99
ΣREY	333	192	356	261	250	185	362	122	207	135

* – bottom ash. SD – standard deviation.

Table S7. Pearson correlation coefficients for the REY content in CFA samples from coal-fired power plants in Serbia.

	Ce	Dy	Er	Eu	Gd	Ho	La	Lu	Nd	Pr	Sm	Tb	Tm	Y
Dy	0.914													
Er	0.886	0.924												
Eu	0.906	0.935	0.884											
Gd	0.943	0.953	0.909	0.955										
Ho	0.907	0.976	0.904	0.946	0.961									
La	0.977	0.927	0.896	0.898	0.937	0.922								
Lu	0.881	0.968	0.890	0.939	0.945	0.940	0.888							
Nd	0.937	0.928	0.924	0.909	0.969	0.921	0.950	0.916						
Pr	0.975	0.878	0.833	0.922	0.953	0.901	0.951	0.863	0.938					
Sm	0.973	0.946	0.875	0.956	0.963	0.940	0.942	0.934	0.925	0.963				
Tb	0.889	0.942	0.934	0.922	0.941	0.938	0.896	0.944	0.941	0.866	0.920			
Tm	0.855	0.920	0.915	0.892	0.896	0.903	0.861	0.939	0.866	0.806	0.885	0.916		
Y	0.926	0.934	0.903	0.963	0.959	0.952	0.935	0.906	0.932	0.935	0.930	0.884	0.878	
Yb	0.916	0.955	0.921	0.942	0.969	0.960	0.888	0.936	0.940	0.914	0.948	0.933	0.888	0.936

$p < 0.05$

Table S8. Enrichment factor (EF) for REY in CFA.

CFA	Ce	Dy	Er	Eu	Gd	Ho	La	Lu	Nd	Pr	Sm	Tb	Tm	Y	Yb
CFA1	3.2	3.9	4.3	3.5	4.0	3.1	3.5	3.8	3.5	2.6	3.4	4.1	3.9	3.1	3.5
CFA2	2.3	2.7	3.4	2.6	2.8	2.7	2.2	1.9	2.3	1.6	2.3	3.6	2.7	2.0	2.6
CFA3	2.5	3.7	3.4	2.8	3.4	3.1	2.7	3.7	2.8	2.0	2.8	3.3	3.8	3.0	3.1
CFA4	2.3	2.4	3.0	2.0	2.4	1.9	2.2	1.9	2.2	1.6	2.1	2.5	3.4	2.1	2.3
CFA5	2.0	2.4	3.0	2.3	2.4	1.9	1.9	1.8	2.2	1.6	2.1	2.1	2.3	2.1	2.4
CFA6	2.0	1.9	2.6	1.8	2.0	1.3	1.6	1.1	1.7	1.2	1.7	1.4	1.1	1.9	2.0
CFA7	3.1	4.7	3.9	3.6	4.4	3.9	3.2	4.0	3.5	2.5	3.6	4.2	4.2	3.6	3.9
CFA8	1.8	2.1	2.3	2.6	2.6	1.4	1.5	2.3	1.6	1.1	2.2	1.8	3.2	1.6	1.7
CFA9	1.5	1.7	1.9	1.7	1.7	1.3	1.3	1.6	1.5	1.2	1.5	1.4	1.6	1.7	1.9
CFA10	0.9	1.2	1.1	0.9	1.3	1.1	0.9	0.5	1.1	0.7	0.8	0.7	0.4	0.9	1.2

Table S9. Geoaccumulation index (I_{geo}) for REY in CFA.

CFA	Ce	Dy	Er	Eu	Gd	Ho	La	Lu	Nd	Pr	Sm	Tb	Tm	Y	Yb
CFA1	0.33	0.64	0.77	0.49	0.64	0.30	0.46	0.59	0.46	0.02	0.43	0.70	0.64	0.30	0.46
CFA2	-0.36	-0.17	0.20	-0.21	-0.11	-0.17	-0.47	-0.63	-0.41	-0.86	-0.36	0.26	-0.17	-0.58	-0.18
CFA3	0.36	0.94	0.82	0.55	0.83	0.68	0.49	0.95	0.53	0.06	0.57	0.78	1.00	0.65	0.68
CFA4	0.10	0.13	0.47	-0.10	0.18	-0.17	0.04	-0.22	0.02	-0.43	-0.01	0.19	0.64	-0.07	0.08
CFA5	-0.03	0.17	0.53	0.12	0.20	-0.17	-0.15	-0.22	0.07	-0.38	0.01	0.03	0.15	-0.01	0.19
CFA6	-0.34	-0.38	0.04	-0.45	-0.30	-0.92	-0.61	-1.22	-0.53	-1.04	-0.55	-0.79	-1.17	-0.39	-0.32
CFA7	0.42	0.99	0.75	0.62	0.90	0.72	0.45	0.78	0.58	0.10	0.63	0.86	0.83	0.60	0.74
CFA8	-0.96	-0.75	-0.66	-0.45	-0.45	-1.33	-1.28	-0.63	-1.14	-1.70	-0.71	-1.00	-0.17	-1.17	-1.08
CFA9	-0.27	-0.06	0.02	-0.10	-0.13	-0.47	-0.51	-0.22	-0.28	-0.58	-0.31	-0.40	-0.17	-0.14	0.04
CFA10	-1.03	-0.58	-0.65	-0.91	-0.39	-0.65	-0.92	-1.95	-0.71	-1.25	-1.19	-1.39	-2.17	-0.92	-0.56
EU	0.06	0.26	0.35	0.34	0.63	-0.05	-0.05	0.37	0.47	0.07	0.29	0.61	0.15	0.07	0.41
UK	0.37	0.54	0.43	0.62	0.67	0.50	0.36	0.45	0.40	0.34	0.58	0.51	0.57	0.57	0.56
USA	0.56	0.93	0.89	1.03	1.12	0.86	0.52	0.90	0.58	0.56	0.84	0.88	1.02	1.03	0.96
China	0.90	0.95	0.91	0.74	1.23	0.82	0.86	0.95	0.89	0.84	0.96	0.93	1.00	0.87	1.00
SA	0.82	0.79	0.69	0.74	0.95	0.59	0.91	0.59	0.70	0.77	0.85	0.68	0.64	0.88	0.51
India	0.53	0.70	0.42	0.51	0.67	0.41	0.30	0.56	0.17	0.32	0.83	0.53	0.50	0.21	0.65
World	0.73	0.91	1.00	0.79	1.17	0.75	0.65	0.78	0.81	0.67	0.90	0.93	1.00	0.89	0.98

Table S10. Potential risk index (RI) for HMs in CFA.

CFA	As	Cd	Co	Cr	Cu	Hg	Mn	Ni	Pb	Zn	RI
CFA1	154	483	4.7	3.6	14	63.2	0.6	11.3	26	0.8	762
CFA2	135	427	4.2	4.8	18	84.8	0.5	12.8	17	0.9	705
CFA3	164	547	6.6	4.6	14	88.0	0.8	15.9	31	1.1	872
CFA4	97	530	4.7	5.9	11	48.0	0.7	20.0	29	0.8	746
CFA5	685	913	6.0	1.9	14	68.8	0.7	7.6	39	3.1	1740
CFA6	132	563	4.9	2.6	18	32.0	0.6	7.6	21	0.9	783
CFA7	283	900	8.1	3.2	19	196.0	0.6	10.7	36	2.4	1459
CFA8	95	433	4.3	4.0	12	80.0	0.4	8.2	10	0.9	649
CFA9	82	577	5.1	2.3	19	40.8	0.8	6.9	21	0.8	756
CFA10	61	483	4.6	2.1	16	8.0	0.9	6.0	16	0.4	598

Table S11. Individual risk index (Er) for HMs and REY in CFA.

REY, HM	Er
Cd	586
As	189
Hg	71.0
Pb	24.7
Cu	15.6
Ni	10.7
Co	5.3
Cr	3.5
Zn	1.2
Mn	0.7
Lu	29.2
Tm	17.0
Tb	16.1
Eu	15.3
Ho	14.2
Er	9.3
Gd	8.7
Dy	8.7
Yb	8.0
Sm	7.3
Pr	5.3
Nd	2.9
Y	2.9
Ce	1.4
La	1.4

Table S12. Mean hazard quotients (HQ) for REY in CFA.

REY	HQa	HQc
Ce	6.1×10^{-3}	5.6×10^{-2}
La	2.9×10^{-3}	2.7×10^{-2}
Nd	2.7×10^{-3}	2.5×10^{-2}
Y	2.1×10^{-3}	1.9×10^{-2}
Pr	5.2×10^{-4}	4.8×10^{-3}
Gd	4.8×10^{-4}	4.5×10^{-3}
Sm	4.7×10^{-4}	4.4×10^{-3}
Dy	4.6×10^{-4}	4.3×10^{-3}
Er	3.0×10^{-4}	2.8×10^{-3}
Yb	2.2×10^{-4}	2.0×10^{-3}
Eu	1.1×10^{-4}	9.8×10^{-4}
Ho	8.1×10^{-5}	7.5×10^{-4}
Tb	7.7×10^{-5}	7.2×10^{-4}
Tm	3.5×10^{-5}	3.3×10^{-4}
Lu	3.1×10^{-5}	2.9×10^{-4}

Table S13. Mean hazard quotients (HQ) for HMs in CFA.

HM	HQa	HQc
As	4.2×10^{-1}	3.9×10^{0}
Cr	9.0×10^{-2}	7.9×10^{-1}
V	3.9×10^{-2}	3.3×10^{-1}
Pb	3.4×10^{-2}	3.1×10^{-1}
Ni	7.0×10^{-3}	6.5×10^{-2}
Mn	6.5×10^{-3}	5.7×10^{-2}
Ba	3.7×10^{-3}	3.5×10^{-2}
Cd	3.4×10^{-3}	2.9×10^{-2}
Cu	3.0×10^{-3}	2.8×10^{-2}
Mo	2.8×10^{-3}	2.6×10^{-2}
Co	1.3×10^{-3}	1.2×10^{-2}
Hg	1.2×10^{-3}	1.1×10^{-2}
Zn	3.8×10^{-4}	3.5×10^{-3}

Table S14. Individual mean cancer risk (CR) for HMs in CFA.

HM	CRa	CRc
As	3.8×10^{-5}	1.5×10^{-4}
Cr	2.6×10^{-5}	9.9×10^{-5}
Cd	3.0×10^{-6}	1.2×10^{-5}
Pb	2.0×10^{-7}	7.8×10^{-7}
